# The impact of COVID-19 on the management of hypertensive disorders of pregnancy: An assessment of quality of care and outcomes

**DOI:** 10.1177/1753495X251386568

**Published:** 2025-10-28

**Authors:** Celya Tidafi, Annabelle Cumyn, Mandy Malick, Abla Alj, Leena Toban, Elisabeth Gagnon, Samuel Lemaire-Paquette, Marie-Ève Roy-Lacroix, Anne-Marie Côté

**Affiliations:** 1 Centre de recherche du Centre Hospitalier Universitaire de Sherbrooke (CHUS), Montreal, Canada

**Keywords:** Hypertensive disorders of pregnancy, COVID-19, pandemic, pregnancy care, pre-eclampsia, healthcare management, telehealth

## Abstract

**Background:**

Hypertensive disorders of pregnancy (HDP) affect up to 10% of women and require close monitoring to prevent adverse outcomes. The COVID-19 pandemic disrupted in-person care, prompting adaptations in follow-up strategies. This study assesses the pandemic's impact on HDP management at a Quebec tertiary-level health center.

**Methods:**

A retrospective cohort study compared a pandemic group to a prepandemic matched control group of pregnant women with HDP (2015–2020). The primary outcome was the difference in the total number of antenatal follow ups.

**Results:**

Although nonsignificant, total follow ups increased by 11% during the pandemic (RR = 1.11; p = 0.122), driven by a fivefold rise in telephone visits (RR = 5.05; p < 0.001), while in-person visits remained stable. Adverse outcomes rates showed no significant changes.

**Conclusion:**

Increased telehealth use complemented stable in-person care, supporting continuity and flexibility in HDP management. Telehealth holds promise as a supportive tool in prenatal care delivery.

## Introduction

### Background

Hypertensive disorders of pregnancy (HDP) significantly impact maternal, fetal, and neonatal health outcomes globally as they affect up to 10% of women during pregnancy.^1,2^ HDP include chronic hypertension, gestational hypertension, and pre-eclampsia.^2,3^ Studies show a marked increase in the incidence of these conditions across various populations, underscoring the need for robust healthcare strategies to manage the rising trend.^4^

HDP is associated with higher rates of maternal and perinatal mortality and morbidity^3^ including preterm birth and low birth weight. The main complication of HDP is pre-eclampsia which occurs in 2% to 8% of pregnancies and accounts for 10% to 15% of maternal mortality.^4^ Considering the potential mortality and morbidity associated with HDP, a close monitoring is required throughout pregnancy to allow for early diagnosis and to avoid complications. Assessment of HDP includes regular blood pressure monitoring, physical examinations and when clinically indicated, laboratory and fetal tests.^2,5^ Management includes prophylactic use of low-dose aspirin starting between 12 and 16 weeks of gestational age until 36 weeks of gestational age, which is effective in reducing the risk of preterm pre-eclampsia in women identified as high-risk.^3,6^ Most guidelines, including the *Journal of Obstetrics and Gynaecology Canada* (JOGC),^3^ recommend initiating low-dose aspirin early in pregnancy in prevention of pre-eclampsia.^7^ It is also important that women have timely initial access to clinical care without delay.

At the ‘Centre intégré universitaire de santé et des services sociaux de l’Estrie – Centre hospitalier universitaire de Sherbrooke (CIUSSS de l’Estrie - CHUS)’ located in Quebec (Canada), many women with HDP are followed by an Obstetric Medicine specialist in the outpatient setting. These women are usually referred and concurrently followed by either Family Medicine, Obstetrics-Gynecology, or Fetal-Maternal Medicine.

The onset of the COVID-19 pandemic introduced unprecedented disruptions to healthcare systems worldwide, compelling a shift toward virtual healthcare delivery.^8^ This transition was particularly crucial for the management of chronic conditions such as HDP, where continuous monitoring is essential.^9,10^ Moreover, innovations in remote monitoring and home blood pressure monitoring have shown promising results in managing HDP, enhancing patient safety and reducing the frequency of hospital visits, which is crucial during pandemics and in areas with limited healthcare access.^5,11^ During the COVID-19 pandemic, the local Obstetric Medicine team was forced to adapt and prioritize virtual follow ups with patients as much as possible with instructions to reduce in-person visits. Considering all the changes imposed by the pandemic, our objective is to evaluate its impact on management and outcomes of women with HDP followed by the Obstetric Medicine team.

## Methods

### Study design

We conducted a single center, retrospective cohort study (pandemic group) with matched controls (prepandemic group). The pairing was done based on the gestational age at time of the first visit with the Obstetric Medicine team. The project was approved by ‘Centre intégré de santé et des services sociaux de l’Estrie-Centre Hospitalier Universitaire de Sherbrooke’ research ethics committee with a delegated consent process (exempt from requiring individual patient consent).

### Population

We included pregnant women aged 18 or above, diagnosed with HDP, followed in Obstetric Medicine and/or Maternal-Fetal Medicine (MFM) and who had given birth at the CIUSSS de l’Estrie - CHUS between March 2015 and June 2021. We excluded women who did not have access to a phone and women whose HDP diagnosis occurred during childbirth or during the postpartum period.

### Definitions

We classified HDP as either chronic hypertension, defined as systolic blood pressure (SBP) ≥ 140 mmHg or diastolic blood pressure (DBP) ≥ 90 mmHg diagnosed before pregnancy or before 20^+0^ weeks of gestation, gestational hypertension, defined as SBP ≥ 140 mmHg or DBP ≥ 90 mmHg diagnosed at 20^+0^ weeks of gestation or later or pre-eclampsia.^12^ Pre-eclampsia has a complex definition that involves, according to the most recent definition,^12^ hypertension, with either proteinuria or at least one adverse conditions and/or severe complications in the mother and/or the fetus. We also included in this category participants who were diagnosed with HELLP syndrome, which is defined as a combination of hemolysis, elevated liver enzymes, and low platelet count. A fourth category, named ‘other hypertensive disorders’, includes transient hypertension, white coat hypertension, masked hypertension, as well as women followed for a history of pre-eclampsia in a previous pregnancy or followed for borderline blood pressure, that is a blood pressure < 140/90 mmHg but approaching this threshold and requiring closer monitoring. Any one participant could be categorized in two different categories if multiple diagnoses were made.

### Sample size

As we have no information about the expected number of follow ups during the pandemic period and as literature^13^ presents an average of 16 ± 4 follow ups for conventional care, a power analysis based on a Mann-Whitney U-test comparing the ranks of the two cohorts was performed.

We assumed the number of follow ups would not follow a normal distribution, which justified the use of a nonparametric approach. Initially, based on a significance level of 5% and a power of 80%, a total of 258 women (172 controls and 86 cases) were required to detect a moderate effect size of 0.4 (Cohen's d) using our matching strategy. However, after data collection, only 208 participants met the eligibility criteria (142 controls and 66 cases). With this final sample size, a post hoc power analysis indicated that the minimal detectable effect size increased to 0.44.

### Study conduct

After obtaining the necessary approvals, the institutional database shared a confidential list of potentially eligible women comprising all delivery summaries indicating an HDP during the study period (March 2015–June 2021). Every pregnancy was considered independently, and a woman could be included more than once.

All pregnancies included in the research were assigned a random code. Every woman from the pandemic group was paired with two women from the prepandemic group according to gestational age at time of the initial consultation with the Obstetric Medicine team, being either first trimester (< 13^+0^ weeks of gestational age), second trimester (between 13^+0^ and 28^+6^ weeks of gestational age) or third trimester (≥ 29^+0^ weeks of gestational age).

### Data collection

Raw data were collected from the participants’ medical files regarding basic demographics, maternal and obstetrical characteristics, gestational age at time of the initial consultation with the Obstetric Medicine team, indications for delivery and neonatal outcomes. The prepandemic group was composed of women who gave birth between 15 March 2015 and 15 June 2019. The pandemic group was composed of women who gave birth between 15 March 2020 and 15 June 2021.

### Outcomes

The primary outcome was the difference in the average number of total antenatal follow ups (in-person and by phone) in Obstetric Medicine between the two groups.

Secondary outcomes included the difference in delivery outcomes, neonatal wellbeing and incidence of pre-eclampsia and its complications between women who had their follow up during the pandemic compared to the prepandemic period. It also included the difference in the number of in-person follow ups, phone follow ups, laboratory assessments, hospitalizations, ultrasound, and nonstress tests, respectively, between the pre- and pandemic periods. All nonstress tests (NSTs) were performed at the hospital; no NSTs were conducted remotely during the study period.

### Statistical analysis

Data analysis was performed using SPSS v.28 software (IBM Corp., Armonk, NY).

For descriptive statistics, dichotomous and categorical variables were reported in frequency and percentage. Continuous variables with normal deviation were reported as means with standard deviation and those with an asymmetric distribution were reported as medians with interquartile deviation. The normality of the variables was assessed on a histogram basis.

To compare the different demographic and descriptive characteristics of our two paired groups we used the Chi-square association test for dichotomous and categorical variables. For theoretical frequencies below 5, the exact Fisher test was preferred. For continuous variables, the Student t-test was used when the data is normally distributed, otherwise the Mann-Whitney nonparametric test was used.

To assess the primary outcome, a mixed Poisson regression model was used to compare the number of follow ups in each group taking into account gestational age at time of the first visit and duration of follow up with the Obstetric Medicine team. Follow-up rates per unit of time has been considered to account for the fact that participants have not all been followed for the same period of time. If a woman was included more than once in our study for two separate pregnancies, we also considered this effect in our analysis by adding a random effect at the patient level, hence considering correlation between a patient's repeated measures. The effect sizes will be presented in rate ratios with their 95% confidence interval.

For secondary objective analyses, a mixed Poisson regression model was done for each of the variables of interest according to the nature of the variables. A significant threshold of 5% and a bilateral testing approach has been considered.

## Results

### Population characteristics

This study included a total of 208 pregnancies, accounting for 10 women who had two pregnancies during the observation period. Overall, 142 pregnancies were followed prepandemic and 66 per pandemic. Detailed demographic and clinical characteristics are summarized in Table 1.

**Table 1.** Characteristics of pregnancies in the prepandemic and pandemic groups.

Description	Overall (n = 208) n (%)	Prepandemic (n = 142) n (%)	Pandemic (n = 66) n (%)	P-value
*Demography and participants description*				
Caucasian	175 (84.5%)	117 (82.4%)	58 (89.2%)	0.207
Gestational age at inclusion (weeks) (average +/− sd)	19.0 +/− 10.7	18.5 +/− 10.6	20.1 +/−10.8	0.247
Maternal age (years) (average +/− sd)	31.0 +/− 5.1	30.9 +/− 5.3	31.3 +/− 4.7	0.572
Primipara	73 (35.1%)	59 (41.5%)	14 (21.2%)	0.004
Body mass index (BMI) (kg/m^2^) (average +/− sd)	33.4 +/− 9.2	33.7 +/− 9.4	32.9 +/− 8.8	0.561
*Comorbidities*				
Endocrine disease	109 (52.4%)	77 (54.2%)	32 (48.5%)	0.440
Renal disease	13 (6.3%)	6 (4.2%)	7 (10.4%)	0.120
Cardiovascular disease	12 (5.3%)	7 (4.9%)	5 (7.6%)	0.525
Bleeding disorders	11 (5.3%)	8 (5.6%)	3 (4.5%)	1.00
Immunological disease	6 (2.9%)	3 (2.1%)	3 (4.5%)	0.384
History of pre-eclampsia	50 (24.0%)	9 (16.1%)	9 (36.0%)	0.515
Current tobacco use	21/200 (10.5%)	13/ 135 (9.6%)	8/65 (12.3%)	0.563
In vitro fertilization	11/207 (5.3%)	7 (4.9%)	4/65 (6.2%)	0.744
Multiple pregnancy	19 (9.1%)	19 (13.4%)	0	0.002
Congenital malformations	5/207 (2.4%)	3/14 (1.8%)	2/65 (3.1%)	0.650
*Antenatal care*				
Participant already on antihypertensive medication at 1^st^ visit	73 (35.1%)	56 (39.4%)	17 (25.8%)	0.054
Aspirin use during pregnancy	136 (65.4%)	90 (63.4%)	46 (69.7%)	0.373
Providers for antenatal follow up*				
Maternal-fetal medicine	160 (76.9%)	110 (77.5%)	50 (75.8%)	0.786
OBGYN	110 (52.9%)	86 (60.6%)	24 (36.4%)	0.001
Family medicine*	105 (50.5%)	69 (48.6%)	36 (54.4%)	0.424
*Initial HDP diagnosis*				
Chronic hypertension	122 (58.7%)	84 (59.2%)	38 (57.6%)	0.830
Gestational hypertension	54 (26.0%)	33 (23.2%)	21 (31.8%)	0.189
Pre-eclampsia including HELLP	18 (9.7%)	12 (8.5%)	6 (9.1%)	0.879
Other hypertensive disorders	51 (24.5%)	38 (26.8%)	13 (19.7%)	0.270
*Final HDP diagnosis*				
Chronic hypertension	125 (60.1%)	88 (62.0%)	37 (56.1%)	0.418
Gestational hypertension	66 (31.7%)	41 (29.8%)	25 (37.9%)	0.194
Pre-eclampsia including HELLP	45 (21.6%)	33 (23.2%)	12 (18.2%)	0.410
Other hypertensive disorders	50 (24.0%)	36 (25.4%)	14 (21.2%)	0.515

*Some participants were followed by more than one care provider during their pregnancies.

Regarding comorbidities, endocrine diseases were present in about half of the cohort (52.4%*)* and remained similar in the prepandemic and pandemic group (p = 0.445). The use of aspirin during pregnancy was common, involving 65.4% of the total study population, with no significant variation between the two groups (p = 0.373). Chronic hypertension was diagnosed in 60.1% of cases overall and was the most frequent final diagnosis among hypertensive disorders.

Regarding follow ups seen by Obstetric Medicine, there were significantly less pregnancies followed by the OBGYN clinic during the pandemic (36.4% vs. 60.6%, p < 0.001). On the other hand, the proportions of other antenatal care providers were similar for both periods. There was a nonsignificantly greater number of participants already on antihypertensive medication at the first Obstetric Medicine visit in the prepandemic group (39.4%) compared to the pandemic group (25.8%) (p = 0.054).

### Antenatal follow-up patterns

The primary outcome of the study was the difference in the average number of total antenatal follow ups with the Obstetric Medicine team, which increased by 11% in the pandemic group (median [Q1; Q3] = 4.0 [2.0; 8.0]) compared to the prepandemic group (median [Q1; Q3] = 5.0 [3.0; 8.0]). However, this difference was not statistically significant (Risk Ratio (RR) = 1.11; 95% confidence interval (CI) [0.97; 1.25]; p = 0.122). Notably, there was a significant shift toward virtual follow ups during the pandemic period, with a fivefold increase in phone follow ups (RR = 5.05; 95% CI [2.89; 8.84]; p < 0.001), although no participant in the prepandemic group had a phone follow up (Table 2). The number of in-person follow- ps remained relatively stable (RR = 1.01; 95% CI [0.88; 1.15]; p = 0.881). Results excluding all pregnancies that had their first antepartum visit prior to 15 March 2020 and are in the pandemic group (n = 10) are presented in Appendix 1.

**Table 2.** Antenatal management prepandemic versus pandemic group.

Description	Prepandemic Median [Q1; Q3] (n = 142)	Pandemic Median [Q1; Q3] (n = 66)	RR (95% CI)	P-value
Number of follow ups				
Total	5.0 [3.0; 8.0]	4.0 [2.0; 8.0]	1.11 (0.97–1.25)	0.122
Phone	0.0 [0.0; 0.0]	0.0 [0.0; 1.0]	5.05 (2.89–8.84)	< 0.001
In person	4.5 [2.0; 8.0]	4.0 [1.0; 7.3]	1.01 (0.88–1.15)	0.881
Number of blood tests	9.0 [6.0; 13.0]	7.0 [5.0; 11.0]	1.00 (0.90–1.11)	0.995
Number of fetal ultrasounds	5.0 [3.0; 8.0]	4.5 [2.0; 7.0]	1.11 (0.98–1.27)	0.106
Number of nonstress test per pregnancy	3.0 [1.0; 5.0]	2.0 [0.0; 3.3]	0.91 (0.76–1.10)	0.319
Number of antenatal hospitalizations	0.0 [0.0; 1.0]	0.0 [0.0; 0.0]	0.73 (0.41–1.28)	0.269

### Testing, delivery, and neonatal outcomes

The number of fetal ultrasounds did not change significantly during the pandemic (RR = 1.11; 95% CI [0.98; 1.27]; p = 0.106), although it did slightly decrease. There was a nonsignificant decrease in the number of antenatal hospitalizations (RR = 0.73; 95% CI [0.41; 1.28]; p = 0.269) (prepandemic [min–max] [0–3]; pandemic [0–2]) and a similar trend for nonstress tests (RR = 0.91; 95% CI [0.76; 1.10]; p = 0.319) in the pandemic group (Table 2).

Delivery and neonatal wellbeing outcomes between the two groups are presented in Table 3. A significant increase in induction of labor in the pandemic group (60.6%) compared to the prepandemic group (45.8%, p = 0.046) was observed. There was also a significant rise in birth weights in the pandemic group, with a median birth weight of 3138 g compared to 2940 grams in the prepandemic group (p = 0.029). Preterm delivery rates, caesarean sections, or neonatal intensive care admissions remained similar in both groups.

**Table 3.** Delivery and neonatal outcomes prepandemic and pandemic groups.

Description	Prepandemic (n = 142) n (%)	Pandemic (n = 66) n (%)	P-value
Delivery outcomes			
Delivery < 37 weeks	45 (31.7%)	14 (21.2%)	0.119
Delivery < 34 weeks	13 (9.2%)	5 (7.6%)	0.706
Induction	65 (45.8%)	40 (60.6%)	0.046
Vaginal delivery	78 (54.9%)	43 (65.2%)	0.164
Instrumental	7 (4.9%)	4 (6.1%)	0.746
Caesarean section (C-section)	63 (44.4%)	23 (34.8%)	0.195
Elective C-section	32 (22.5%)	10 (15.2%)	0.217
Emergency C-section	31 (21.8%)	13 (19.7%)	0.726
Neonatal wellbeing			
APGAR score at 1 min (median [Q1; Q3])	8 [6; 9]	8 [7; 9]	0.893
APGAR score at 5 min (median [Q1; Q3])	9 [8; 10]	9 [8; 9]	0.353
Birthweight (median [Q1; Q3])	2940 [2545; 3315]	3138 [2780; 3494]	0.029
Admission to neonatal care	36 (25.4%)	9 (13.6%)	0.056
Mortality	2 (1.4%)	0 (0%)	1.000

To account for potential confounders, we conducted multivariable analyses adjusting for gestational age at delivery, maternal age, multiple pregnancies, and history of pre-eclampsia (Appendix 2). After adjustment, there were no significant differences between the pandemic and prepandemic groups in APGAR (Appearance, Pulse, Grimace, Activity and Respiration) scores at 1 or 5 minutes, birthweight, preterm birth <37 weeks, or induction of labor. However, the adjusted mean birthweight difference between groups approached statistical significance, with a higher birthweight in the pandemic group (adjusted mean difference = 126.24 g; 95% CI: −9.97 to 262.45; p = 0.069). Admission to neonatal care was also lower in the pandemic group (adjusted odds ratio (aOR) = 0.37; 95% CI: 0.10–1.11; p = 0.099), though this did not reach statistical significance.

### Pre-eclampsia and related complications

The occurrence of pre-eclampsia and related complications between the prepandemic group and the pandemic group was also assessed (Table 4). Overall, 18 women were diagnosed with pre-eclampsia during their pregnancy (8.7%), 12 of them in the prepandemic group (8.5%) and 6 of them in the pandemic group (9.1%). Fewer instances of increased liver enzymes and thrombocytopenia related to pre-eclampsia were observed prepandemic, though these findings did not reach statistical significance. Unique to the pandemic group, one patient experienced both acute kidney injury (AKI) and required intensive care unit (ICU) admission, another had an isolated AKI, and a third had a placental abruption. These complications occurred in three distinct individuals. There was no neonatal convulsion nor mortality in either group. There was no significant difference between the two groups regarding the other neonatal complications.

**Table 4.** Maternal and neonatal complications related to pre-eclampsia.

Description	Prepandemic (n = 12) n (%)	Pandemic (n = 6) n (%)	P-value
Maternal complications related to pre-eclampsia			
Increased liver enzymes	6 (50.0%)	0 (0%)	0.054
Thrombocytopenia	4 (33.3%)	0 (0%)	0.245
Acute kidney injury	0 (0%)	2 (33.3%)	0.098
Intensive care unit admission	0 (0%)	1 (16.7%)	0.333
Placental abruption	0 (0%)	1 (16.7%)	0.333
Postpartum hemorrhage	0 (0%)	0 (0%)	1.000
Eclampsia	0 (0%)	0 (0%)	1.000
Retinal detachment	0 (0%)	0 (0%)	1.000
Pulmonary edema	0 (0%)	0 (0%)	1.000
Stroke	0 (0%)	0 (0%)	1.000
Neonatal complications related to pre-eclampsia			
Admission to neonatal care	5 (41.7%)	3 (50.0%)	1.000
Respiratory support	3 (25%)	2 (33.3%)	1.000
Small for gestational age	1 (8.3%)	2 (33.3%)	0.245
Neonatal convulsions	0 (0%)	0 (0%)	1.000
Mortality	0 (0%)	0 (0%)	1.000

## Discussion

### Main findings

Our study found that the overall number of antenatal follow ups was not significantly affected by the pandemic, with a notable shift toward remote consultations. The significant increase in phone follow ups by fivefold (RR = 5.05; 95% CI [2.89; 8.84]; p < 0.001) indicates that telehealth was integrated as an additional tool to support continuity of care when in-person visits were restricted.^8^ Given that in-person visits remained stable (RR = 1.01; 95% CI [0.88; 1.15]; p = 0.881), this suggests that telehealth did not replace in-person care but rather complemented it, allowing for flexibility in patient follow up without compromising access to necessary in-person evaluations. This finding is consistent with the literature indicating that remote monitoring and telehealth have been crucial in managing high-risk pregnancies during the COVID-19 pandemic, enhancing patient safety, and reducing the need for hospital visits.^8,14^

Despite the increase in remote consultations, we observed that the number of in-person visits remained stable (RR = 1.01; 95% CI [0.88; 1.15]; p = 0.881) as well as the number of tests including nonstress tests and fetal ultrasounds with a nonsignificant decrease. This suggests that essential in-person care continued to be provided when necessary. This balance between remote and in-person care likely helped in maintaining effective management of HDP during the pandemic.

The study also showed a higher birthweight in the pandemic group, which remained after adjusting for gestational age at delivery, maternal age, multiple pregnancies, and history of pre-eclampsia. Although the difference did not reach statistical significance (adjusted mean difference = 126.24 g; 95% CI: −9.97 to 262.45; p = 0.069), the trend is notable and may reflect better growth outcomes or fewer preterm deliveries during the pandemic. While admission to neonatal care was less frequent in the pandemic group, this association also did not reach statistical significance after adjustment. These findings suggest that the observed trends in improved neonatal outcomes were not fully explained by baseline differences in patient characteristics.

### Interpretation

The resilience of healthcare systems during the COVID-19 pandemic, particularly in managing hypertensive disorders of pregnancy (HDP), highlights a pivotal shift toward integrating telehealth into routine care. The fivefold increase in phone follow ups aligns with global trends where providers leveraged telehealth to sustain engagement and continuity of care.^14,15^ Notably, this study underscores that despite the substantial shift to telehealth, the quality of care, measured through the stable rate of in-person visits and antenatal management outcomes, was maintained. However, current guidelines no longer recommend favoring virtual follow ups.^15,16^ A patient-centered, blended approach combining in-person and remote consultations has demonstrated utility.^17^ This strategy not only supports care management but also represents a socially responsible practice, improving adherence among patients facing barriers such as transportation, work, family obligations, or financial constraints.

The stability in severe maternal outcomes may partly reflect enhanced surveillance enabled by telehealth, ensuring ongoing monitoring and early HDP complication detection.^11,18,19^ Stable in-person visit rates reflect providers’ commitment to essential services, consistent with reports from other regions emphasizing adaptations to preserve critical care.^10^ Continued in-person care likely contributed to the observed stability in maternal and neonatal outcomes. Interestingly, our data show a nonsignificant increase in the number of follow ups during the pandemic (RR = 1.11; 95% CI [0.97; 1.25]; p = 0.122). This may suggest a potential discomfort^20^ or uncertainty with remote follow ups early in the pandemic, leading to a possible overcompensation in the frequency of in-person follow ups. It may also reflect improved access for patients challenged by long travel distances. This hypothesis highlights how the initial transition to remote care may have prompted increased follow ups to ensure safety and adherence.

The stable number of antenatal hospitalizations and nonstress tests suggests that the reallocation of resources and prioritization of care did not adversely affect the overall quality of care. This finding is supported by research indicating that many healthcare systems managed to maintain or even enhance the quality of care for pregnant women during the pandemic through targeted resource allocation and prioritization of high-risk cases.^9,10^

Complications related to pre-eclampsia were overall infrequent. Acute renal failure, placental abruption, and intensive care unit admissions all occurred during the pandemic period. While these complications were observed, it is important to note that no definitive conclusions can be drawn due to the very low incidence.

### Strengths and limitations

This is a small, single center study conducted in Canada with universal coverage of healthcare. The results cannot necessarily be extended to centers with other models of care. In contrast, fragmented healthcare systems, may face additional barriers to equitable telehealth implementation. In the United States, women from marginalized populations often face reduced access to telehealth due to limited digital literacy, lack of privacy, and systemic mistrust, factors that are not always adequately addressed in care models designed for high-resource environments.^21^ Similar concerns have been raised regarding the role of insurance-based access and fragmented care delivery in exacerbating disparities in maternal telehealth utilization.^22^ In low- and middle-income countries (LMICs), digital tools such as mobile health platforms have improved access to maternal healthcare, but uptake remains dependent on digital readiness, internet infrastructure, and literacy.^23^ Although many women express willingness to use telemedicine, practical barriers such as affordability of devices, cultural familiarity with technology, and network coverage persist.^24^ Moreover, challenges such as provider digital training and language inclusivity continue to limit equitable integration of telehealth services in antenatal care.^25^ These findings emphasize the need for contextualized telehealth strategies and prioritization of digital inclusion to ensure equitable access to maternal care across diverse healthcare systems.

This study classifies the participants in the pandemic group based on delivery date rather than conception date. As a result, women who gave birth early in the pandemic (March–April 2020) may have received most of their antenatal care before the implementation of COVID-19-related restrictions, potentially minimizing their exposure to changes in care delivery. This could have led to an underestimation of the pandemic's true impact. Although this limitation may theoretically bias the results toward the null and lead to an underestimation of the pandemic's true impact, we believe its effect on the main findings is minimal. Notably, no participants had telephone follow ups prior to the pandemic, reducing the risk of misclassification for our primary exposure. Additionally, most clinical variables involved small numbers of events, further limiting the potential for distortion. For transparency, we conducted a sensitivity analysis excluding women in the pandemic cohort who were likely followed predominantly before March 2020. Results of this analysis, presented in the Appendix, are consistent with the main findings. In fact, the total number of antenatal follow ups remained significantly higher in the pandemic group (RR = 1.23; 95% CI [1.07–1.42]; p = 0.004), driven by a marked increase in phone follow ups (RR = 6.76; 95% CI [3.70–12.37]; p < 0.001), along with higher rates of blood tests and fetal ultrasounds. This reinforces the robustness of our primary findings. However, classification by delivery date ensured consistency in data collection, as delivery records were systematically available, unlike precise conception or early pregnancy care data.

Another limitation is that the final sample size did not reach the number estimated in the initial power calculation. A total of 258 participants was deemed necessary to detect a moderate effect size with adequate statistical power. However, for the selected study periods and defined eligibility criteria, no additional patients meeting inclusion criteria were available in the institutional database. This smaller sample size may have reduced the ability to detect small but potentially meaningful differences between the groups.

While telehealth has proven beneficial, its application is not without limitations. The variability in access to technology across different socioeconomic groups can lead to disparities in healthcare access, as highlighted by studies and guidelines noting that lower socioeconomic status is linked to reduced access to telehealth services.^26–28^ This is crucial for HDP management, where delayed or insufficient care can lead to adverse outcomes. Therefore, future strategies should focus on making telehealth more inclusive and accessible to all patient demographics.

## Conclusion

In conclusion, our study highlights the critical role of telehealth in managing HDP during the COVID-19 pandemic, demonstrating that healthcare systems can adapt rapidly to ensure continued quality of care under challenging circumstances. The findings underscore the potential of telehealth as a complementary tool in prenatal care, which can be integrated into future healthcare practices beyond the pandemic context.^29^ Future studies should include more women, different models of antenatal care, and a greater diversity of woman. They could also explore women's perspectives on mixed approaches to the follow up of HDP in pregnancy.

## Appendix 1. Antenatal management prepandemic versus pandemic group excluding early pandemic pregnancies*

**Table:**

Description	Prepandemic Median [Q1; Q3] (n = 142)	Pandemic Median [Q1; Q3] (n = 56)	RR (95% CI)	P-value
Number of follow ups				
Total	5.0 [3.0; 8.0]	3.0 [2.0; 8.0]	1.23 (1.07–1.42)	0.004
Phone	0.0 [0.0; 0.0]	0.0 [0.0; 1.0]	6.76 (3.70- 12.37)	< 0.001
In person	4.5 [2.3; 8.0]	3.0 [1.0; 7.3]	1.11 (0.95- 1.28)	0.183
Number of blood tests	9.0 [6.0; 13.0]	6.5 [5.0; 11.0]	1.13 (1.01–1.26)	0.040
Number of fetal ultrasounds	5.0 [3.0; 8.0]	3.0 [2.0; 7.0]	1.21 (1.05–1.40)	0.011
Number of nonstress test per pregnancy	3.0 [1.0; 5.0]	2.0 [0.0; 3.0]	1.06 (0.86–1.30)	0.605
Number of antenatal hospitalizations	0.0 [0.0; 1.0]	0.0 [0.0; 0.0]	0.83 (0.45–1.52)	0.583

* Excluding all pregnancies that had their first antepartum visit prior to 15 March 2020 and are in the pandemic group (n = 10).

## Appendix 2. Adjusted* delivery and neonatal outcomes prepandemic and pandemic groups.

**Table:**

Description	Adjusted odds ratio (aOR) [95% CI]	Adjusted mean difference (aMD) [95% CI]	P-value
Delivery outcomes			
Delivery < 37 weeks	0.33 [0.01–5.88]	-	0.440
Induction	1.55 [0.82–2.97]	-	0.178
Neonatal wellbeing			
APGAR score at 1 minute	-	0.17 [−0.47–0.82]	0.601
APGAR score at 5 minutes	-	−0.07 [−0.46–0.32]	0.722
Birthweight	-	126.24 [−9.97–262.45]	0.069
Admission to neonatal care	0.37 [0.10–1.11]	-	0.099

*Adjusted for gestational age at delivery, maternal age, multiple pregnancies and history of pre-eclampsia.
